# High Frequency of Depression in Advanced Cancer with Concomitant Comorbidities: A Registry Study

**DOI:** 10.3390/cancers17071214

**Published:** 2025-04-03

**Authors:** Peter Strang, Torbjörn Schultz

**Affiliations:** 1 Department of Oncology-Pathology, Karolinska Institutet, Stockholms Sjukhem Foundation, Mariebergsgatan 22, SE 112 19 Stockholm, Sweden; 2 Research and Development Department, Stockholms Sjukhem Foundation, Mariebergsgatan 22, SE 112 19 Stockholm, Sweden; torbjornschultz@gmail.com

**Keywords:** depression, cancer, frailty, multimorbidity, female, ICD 10

## Abstract

**Background/objectives:** Depression is a common complication of cancer and is associated with distress and reduced participation in medical care. The prevalence is still uncertain in advanced cancer due to methodological problems. Our aim was to study depression in the last year of life and related variables. **Methods:** We used an administrative database and analyzed clinically verified diagnoses of depression during the last year of life for 27,343 persons (nursing home residents excluded) and related the data to age, sex, socioeconomic status on an area level (Mosaic system), and frailty risk as calculated by the Hospital Frailty Risk Score (HFRS). T-tests, chi-2 tests, and binary logistic regression models were used. **Results:** During the last year of life, a clinical diagnosis of depression was found in 1168/27,343 (4.3%) cases and more frequently seen in women (4.8% vs. 3.8%, *p* = 0.001), in the elderly aged 80 years or more, *p* = 0.03, and especially in persons with a frailty risk according to the HFRS, with rates of 3.3%, 5.3% and 7.8% in the low-risk, intermediate and high-risk groups, respectively (*p* < 0.001), whereas no differences were found based on socioeconomic status. In a multiple logistic regression model, being female (aOR 1.30, 95% CI 1.16–1.46) or having an intermediate (1.66, 1.46–1.88) or high frailty risk (2.57, 2.10–3.14) retained the predictive value (*p* < 0.001, respectively). **Conclusions:** Depression is more common in women and, above all, in people with multimorbidity. Depression affects the amount of health care needed, including the need for psychiatric care. Therefore, it should be included in clinical decision-making, especially as depression is associated with poorer prognosis in cancer.

## Simple Summary

Depression is a common complication of cancer and is associated with distress, suffering, and reduced participation in medical care. Although many studies have been published, the prevalence is still uncertain in advanced cancer. This is due to methodological problems, convenience sampling, problems with the definition of depression in cancer, and publication bias, which favors small studies with high prevalences. Our aim was to calculate the one-year prevalence of ICD-10-verified depression in the last year of life and relate the frequency to clinical variables. We showed in a homogeneous population of 27,434 deceased patients that the one-year prevalence was 4.3% and higher in certain cancer types, such as hematologic malignancies. Higher depression rates were seen in females and in persons with a frailty risk or comorbidities. Depression was associated with more emergency room visits and psychiatric consultations. Any depression should be included in clinical decision-making.

## 1. Introduction

Cancer is a potentially life-threatening disease, and even when the prognosis is very favorable, for example, as regards thyroid cancer, a life crisis often occurs [1]. Such a crisis is characterized by symptoms such as shock, disbelief, insomnia, and anxiety but also dysphoric moods or sadness [2]. However, whereas sadness is a natural and often transient response to a negative and distressing event, depression is characterized by a more severe and persistent condition.

In routine clinical care, a diagnosis of major depression is made by a physician based on accepted, formal psychiatric criteria. However, in cancer studies, “depression” is often vaguely defined and can include everything from sadness, mood disturbances, adjustment disorders to actual depression. In addition, assessments have been made in different ways: sometimes only a single question in the VAS format (0 to 10) has been applied; in other cases, instruments such as the Hospital Anxiety and Depression Scale (HADS) have been used with various cut-off values, while more rarely, clinical assessments have been made based on established criteria for depression, for example, the Diagnostic and Statistical Manual of Mental Disorders (DSM) criteria or the International Statistical Classification of Diseases and Related Health Problems, Tenth Revision (ICD-10) criteria. In fact, more than a hundred different assessment methods have been used in palliative care studies of depressive mood or adjustment disorders; however, these conditions have often been labeled as depression [3]. Moreover, the numbers reported may refer to different phases in the cancer trajectory or to different subgroups that are not necessarily representative of the entire population [4]. For these reasons, the literature provides varying prevalence figures between 1 and 69% [5].

In the case of major depression, the person suffers greatly in the form of fatigue, insomnia, a lack of initiative, loss of appetite, a sense of meaninglessness, and sometimes also suicidal thoughts [6]. Concomitant cancer and depression can also lead to treatment interruption, longer stays in hospital, and even poorer cancer survival [7,8,9,10], although some studies are inconclusive as regards prognosis [11].

The problem with depression diagnosis in cancer is that the DSM and ICD criteria assume that the person is somatically healthy. In the description of the ICD-10 diagnosis of F32 (depressive episode), a part of the verbatim definition reads as follows: “…the patient suffers from lowering of mood, reduction of energy, and decrease in activity. Capacity for enjoyment, interest, and concentration is reduced…” [12]; the definition also mentions, e.g., early awakening, loss of appetite, weight loss, and loss of libido. While these symptoms may indicate severe depression in a physically healthy person, the same problems may be due to tumor progression rather than to depression, in persons with advanced cancer [4]. For this reason, rating scales that include such symptoms may indeed indicate a state of depressed mood, but not necessarily clinical depression [4]. A medical assessment by a physician using ICD-10 criteria is safer but probably also results in lower frequencies.

In previous studies, depression or depressive symptoms have been associated with variables such as certain cancer types, e.g., lung [13] or pancreatic cancer [14,15,16], female sex [17], younger patients [17], and also with symptom burden and proximity to death, with a higher frequency during the last months of life [18]. The latter study indicates that the prevalence of depression may be high at the end of life, but there is a demand for palliative care studies that include homogeneous populations and with evaluations that can be replicated [4].

To conclude, there is a difference between “depressive mood” and transitory adjustment disorder, compared to major depression, which is diagnosed by a physician in routine health care. The aim was, therefore, to study the latter, i.e., the frequency of actual ICD-10 diagnoses of depression during the last year of life in patients with advanced cancer in a comprehensive registry study, and to study which variables are associated with an increased likelihood of a depression diagnosis.

## 2. Materials and Methods

### 2.1. Study Design

This study was conducted on retrospective data from Region Stockholm’s administrative database, which is used for the follow-up of all health care within Stockholm County, covering almost 2.5 million inhabitants.

The manuscript is structured according to the recommendations in Strengthening the Reporting of Observational Studies in Epidemiology (STROBE) [19]. Data were collected for all patients who died of advanced cancer during 2015–2023.

### 2.2. Population

Patients aged over 18 years in ordinary accommodation who died with advanced cancer between 2015 and 2023 were included, except for patients with two or more concomitant main cancer diagnoses. Those who were nursing home residents were also excluded, as they are not representative of the general population.

### 2.3. Data Sources/Measurements and Variables

All data used in this study was retrieved from the administrative database. Depression was identified using the clinical International Classification of Diseases 10th Revision (ICD-10) diagnoses that have been set by doctors in connection with regular health care visits in outpatient or inpatient settings. The following ICD-10 codes were included: F32, F33, and F34.

Other variables used to characterize persons with depression were as follows: age groups, sex, Mosaic as a measure of socioeconomic status on an area level [20], and frailty risk as defined by the Hospital Frailty Risk Score (HFRS) [21]. Emergency room (ER) visits, visits in psychiatric services as well as emergency hospitals as places of death were used as outcome measures.

Mosaic is a commercial measure used to identify limited residential areas with similar socioeconomic characteristics [20,22]. Mosaic group 1 covers the most affluent areas, whereas Mosaic group 3 is typical for less affluent neighborhoods. The Mosaic system is described in detail elsewhere [23].

The HFRS is a proxy measure for frailty measurements [21]. It is not based on clinical assessments in individual patients but on 109 weighted ICD-10 codes frequently seen in persons with frailty (the ICD-10 codes assigned by physicians in routine care were directly retrieved from the administrative database). Consequently, it is not an actual measure of frailty but of calculated frailty risk. As the HFRS is based on registered ICD-10 codes, the frailty risk can be calculated retrospectively based on registered data. The HFRS is divided into three groups: low risk (HRFS < 5), intermediate risk (HFRS 5–15), and high risk (HFRS > 15).

### 2.4. Selection Bias and Dropouts

Since all health care interventions must be reported to the regional database as a basis for the financial reimbursement to the health care unit, the data are complete, except for the human factor (error entries).

### 2.5. Study Size

No power calculations were performed as the total cohort (all deaths) between 2015 and 2023 was included.

### 2.6. Statistical Methods and Missing Data

T-tests, Wilcoxon Rank Sum tests, and chi-square tests were applied to compare the groups. Univariable logistic regression analyses of depression (dependent variable) were performed for age groups, sex, Mosaic, and the HFRS (i.e., independent variables), which were then entered into multivariable logistic regression models if the *p*-value was below *p* = 0.10. C-statistic was used as the main measure of goodness of fit for binary outcomes in our multiple logistic regression models, with the interpretation that a C-statistic value of 0.5 indicates that the model is no better than chance at making a prediction, whereas a value of 1.0 indicates a perfect fit. It was completed with the Hosmer and Lemeshow goodness of fit test, which indicates a good fitting model when its value is greater than 0.05.

There were a few missing data (Mosaic classification of 41 patients), which were not substituted. The SAS 9.4/Enterprise guide 8.2 was used for the statistical analysis.

### 2.7. Ethics

The Regional Ethical Review Authority (EPN 2017/1141-31) approved this study.

## 3. Results

In total, 30,339 patients died of advanced cancer during the years 2015 to 2023, of which 2333 were nursing home residents and therefore excluded. Of the remaining 28,006 patients in ordinary accommodation, 663 had two main cancer diagnoses, and 9 patients had three concomitant diagnoses. These were excluded from further analyses, which left a total of 27,343 patients for the main analysis.

### 3.1. Demographic and Clinical Data

Among the 27,343 patients, 49% were women and 51% were men. The mean age was 72.4 (sd 12.2) years. The number of persons with a verified ICD-10 diagnosis of depression was 1168/27343, i.e., 4.3% (Table 1), and the one-year prevalence did not change significantly from 2015 to 2023, although there was a peak in the first year of the pandemic in 2020, with a rate of 5.3%. The proportion of persons with a diagnosis of depression during their last year of life was higher for those aged 80 years or older (4.7% vs. 4.1%, *p* = 0.03) and in women (4.8% vs. 3.8%, *p* = 0.001). Depression was highly correlated to frailty risk, as calculated with the aid of the HFRS: among persons with a low (HFRS < 5), intermediate (5–15), and high (>15) frailty risk, the rates were 3.3%, 5.3% and 7.8% of depression (*p* < 0.001).

### 3.2. Depression and Cancer Type

The proportion of depression varied with the type of cancer. The highest frequencies were seen in hematologic malignancies and in brain tumors, whereas the lowest frequencies were associated with colo-rectal, pancreatic, and breast cancer (Table 2).

### 3.3. Depression and Health Care Consumption

Among persons with depression, there were more visits to emergency departments (*p* < 0.001) and psychiatric services (*p* < 0.001). They received intravenous cancer treatment less often (*p* < 0.001) but received specialized palliative care to the same extent. Likewise, there was no difference between the groups regarding emergency hospitals as places of death. The results are shown in Table 3.

### 3.4. Regression Analyses

In univariable analyses, older age, female sex, and a higher risk of frailty according to the HFRS were all associated with higher frequencies of depression (Table 4). In a multivariable model, age lost its significance, whereas the adjusted odds ratio (aOR) for women was 1.30 (1.16–1.46, *p* < 0.001). However, the strongest association was seen for frailty risk: for those with an intermediate risk of frailty, the aOR was 1.66 (1.46–1.88, *p* < 0.001), whereas the aOR for those with a high frailty risk was 2.57 (2.10–3.14, *p* < 0.001) (Table 4).

## 4. Discussion

This last-year-of-life study, which included 27,343 deceased persons with advanced cancer, showed that the overall rate of clinical depression, formally diagnosed by physicians in ordinary work, was 4.3% during the last year of life, with higher rates in women and in persons with frailty risk. The diagnosis was set as the main or secondary diagnosis only if depression was of clinically significant importance during an episode of care.

The prevalence was higher in certain cancer types, such as hematologic and CNS malignancies. Socioeconomic Mosaic groups were not related to depression. As regards the amount of care needed, depression was associated with more emergency department visits as well as with care within psychiatric care services during the last year of life. On the other hand, depression was associated with less intensive cancer treatments but still not associated with the receipt of specialist palliative care (SPC).

The prevalence of 4.3% of depression is low compared to many other studies of depression in palliative cancer care [5,24,25,26]. The low one-year prevalence may be partly due to the exclusion of nursing home residents, a group in which the prevalence may be higher. However, when past year prevalences are reported in large epidemiological studies on general populations, similar figures of 4–5% are reported [27,28]. Our low prevalence figures compared with other findings published in palliative cancer studies are probably due to methodological issues. E.g., in a systematic review of depression in adults with cancer, Walker et al. studied 499 potentially eligible studies [29]. Of these, 66 studies were considered relevant, and only 15 out of 66 studies met the quality criteria that were set for the review. Of these 15 studies, only two reported confidence intervals for their estimates. This may explain the great range in the reported depression rates, as only a minority of studies meet reasonable quality criteria.

In one of the few palliative care studies where actual clinical interviews were conducted, the prevalence of depression was 6.7%, i.e., close to our results [30]. In that study, the interviews were performed by a trained psychiatrist with the aid of DSM-IIIR criteria [30]. As reviewed by Janberidze et al., other studies that have found higher frequencies have typically used instruments such as the Hospital Anxiety and Depression Scale (HADS) and Beck Depression Inventory, as well as more unspecific instruments such as the Edmonton Symptom Assessment Scale (ESAS), variants of the European Organization For Research And Treatment Of Cancer (EORTC) QLQ30, the Functional Assessment of Chronic Illness Therapy (FACIT), or visual analog scales, to mention a few [4]. HADS is frequently used in studies, but even when this is the case, data are not always comparable as different authors have used different cut-off values for the identification of depression, which affects the outcome to a great extent [26].

In our data, the prevalence of depression was not evenly distributed among different cancer types, which is partially in agreement with other studies. Previous studies have found higher frequencies, e.g., for gynecologic cancer [17], whereas a meta-analysis found insufficient data to analyze individual cancer types [31]., e.g., lung cancer [29] or head–neck cancer [32], which have often but not always been associated with higher rates of depression, but the rates for these cancer types were close to the mean in our analysis. We do not know the exact reason, but since different conditions such as adjustment disorders, depressed moods, or actual clinical depression can form the basis of the analysis, the outcome can be different in different studies.

In our data, higher rates of depression were associated with being female. As summarized by Waraich et al. in a comprehensive review, the 1-year prevalence rates, in general populations, are consistently 1.5 to 2 times higher for females [28]. However, in studies on advanced cancer, data are contradictory. In some studies, higher rates are seen for women [33,34,35], which is in agreement with our findings, whereas no statistical difference was found in a meta-analysis by Mitchell et al. [31].

As shown in reviews and meta-analyses but also in individual studies, socioeconomic vulnerability is associated with depression [36,37,38,39]. As regards cancer, there is compelling data on the importance of socioeconomic factors with respect to incidence, screening, treatment, and prognosis, but only a limited number of studies on depression. In a recent study, higher levels of education as well as marriage were stated as protective measures against the development of depression [40,41]. In another study, lower income was associated with more depressive symptoms [42]. However, in our data, there was no difference in depression rates between persons living in different socioeconomic Mosaic areas. The reason for this remains unclear.

In our multivariable model, the strongest association was seen between depression and frailty risk, with the highest aOR obtained for those with a high frailty risk according to the HFRS. Although the HFRS is a proxy measure for frailty, the score is not based on actual clinical frailty assessments but on 109 weighted ICD-10 diagnoses commonly seen in persons with frailty [21]. In that sense, the HFRS is effectively a measure of comorbidities, just like the frequently used Charlson Comorbidity Index (CCI) [43], but the HFRS is based on more diagnoses. An association between physical multimorbidity and depression is seen in the general population [44], but also regarding cancer [35,45,46,47,48,49] and cancer survivors [50].

Depression is sometimes considered solely as a separate mental illness, and it is easy to overlook how low mood can express itself in the form of physical symptoms. Still, only limited data are available on the use of somatic health care in people with cancer and co-occurring depression. For this reason, we wanted to investigate whether a formal diagnosis of depression was associated with an increased rate of ER visits, in line with the situation of non-cancer diagnoses [51,52]. It is conceivable that depression can lead to more concern about acute health, leading to more ER visits. On the other hand, a depressed person is passive and possibly more reluctant to seek medical help.

In the data we presented, those with depression had more emergency room (ER) visits during their last year of life, partly in line with other cancer studies; however, these show mixed results [53], e.g., Fond et al. showed that people with cancer and depression had a slightly lower frequency of ER visits in the last month of life, but if hospitalized, their stay was longer [49]. In the study by Ye et al. [54] or Mossman et al. [53], there was a trend toward more ER visits, but the sample sizes were too limited for definite conclusions.

Previous studies have indicated a relationship between depression in cancer and reduced frequency of high-intensity treatments, e.g., admissions to intensive care units (ICU), but a higher likelihood of referral to specialist palliative care (SPC) units [49,53,55]. In our data, depression was associated with a lower likelihood of intensive, intravenous cancer treatments. However, depression was not associated with a higher receipt of SPC, in contrast to some other studies [49,53,55]. This is possibly due to different treatment routines and economic considerations. In Swedish tax-financed health care, SPC is not a last resort but is often used as a support function for active cancer treatment, where the early integration of oncology and palliative care is aimed at for certain individuals.

Finally, our data showed that visits to psychiatric services were relatively common among those with a formal diagnosis of depression; 28% had at least one visit. This finding underlines the need for cooperation between oncology and psychiatry, as psychiatric conditions also occur within hospice and may require psychiatric expertise [56].

### 4.1. Strengths and Limitations

Depression studies in cancer have been criticized because of methodological problems, convenience sampling, problems with the definition of depression in cancer, and publication bias, which favors small studies with high prevalences. Studies also include people at different stages of their cancer in the same study, making the data more heterogeneous. A strength of our study is that all cancer patients who died during 2015–2023 were included (except nursing home residents), and the time period studied was clearly delimited to the last year of life. Moreover, it is a relatively large study including more than 1000 people with clinical depression, and we have used a relatively conservative measure for the diagnosis of depression, namely that it should be based on a formal ICD-10 diagnosis made by physicians in routine health care. These strengths are in line with the conclusions by Mitchell et al., who found an association between high study quality and low prevalence and also between a higher number of studied patients and higher quality [31].

The broad and more non-specific assessments of depressive mood with high prevalence figures in several studies are still valuable because they indicate suffering, that is possibly avoidable. However, a strength of our conservative estimates is that our assessments probably target the group that is most likely to benefit from antidepressants.

An important limitation is that our study is merely designed to detect associations between variables and associations that may or may not have a causal relationship, e.g., we found a significant association between depression and a lower rate of intravenous oncological treatments, but we did not have data to prove whether depression preceded or affected the treatment decision, even if it was probable, from a clinical perspective.

Selecting only people who have a formal depression diagnosis is a limitation if the goal is to identify every person with mild or moderate depressive symptoms, i.e., people who may benefit from social support in situations where antidepressants are not needed.

### 4.2. Future Studies

Since data today are heterogeneous, given that data collection occurs at different phases during the cancer trajectory, and since “depression” in studies can signify anything from depressive symptoms, depressive moods, psychological stress, temporary adjustment disorders, and major depression, studies are needed to sort these concepts and to provide a better picture of actual prevalences. Longitudinal studies could be a way to better understand the progression of depression in advanced cancer patients.

In future studies, artificial intelligence (AI) could also be helpful by improving data collection, analysis, and treatment personalization. As an example, machine learning may be helpful to analyze large datasets, e.g., electronic health records, genetic data, and lifestyle factors. With the aid of cluster analyses, patient subtypes with similar clinical features, symptoms, or even biomarkers could be detected. Furthermore, AI can be used as an aid to predict which interventions (e.g., medication, psychotherapy, digital tools) are likely to be most effective, thereby improving person-centered care.

## 5. Conclusions

In this comprehensive study, we were able to show that depression is more common in women and, above all, in people with multimorbidity. Depression affects the amount of health care a person needs, including the need for psychiatric care. Therefore, it should be included in clinical decision-making, especially as depression is associated with poorer prognosis in cancer.

## Figures and Tables

**Table 1:** Demographic and clinical data in 27,343 deceased patients of which 1168 (4.3%) were diagnosed with depression in the last year of life.

Variable	Total (%) 27,343	Depression ^1^ *n* = 1168 (4.3%)	No Depression ^1^ *n* = 26,175 (95.7%)	Chi-Square (DF)	*p*-Value
Age groups				4.5 (1)	0.03
18–79 years, *n* (%)	19,362 (71)	795 (4.1)	18,567 (95.9)		
80 years or more, *n* (%)	7951 (29)	343 (4.7)	7608 (95.3)		
Sex				14.5 (1)	<0.001
Women, *n* (%)	13,328 (49)	633 (4.8)	12,695 (95.2)		
Men, *n* (%)	14,015 (51)	535 (3.8)	13,480 (96.2)		
Mosaic groups ^2^				0.3 (2)	0.86
Group 1, *n* (%)	7416 (27)	324 (4.4)	7092 (95.6)		
Group 2, *n* (%)	11,277 (41)	481 (4.3)	10,796 (95.7)		
Group 3, *n* (%)	8650 (32)	363 (4.2)	8287 (95.8)		
HFRS ^3^				116.0 (2)	<0.001
Group 1, *n* (%)	15,713 (58)	512 (3.3)	15,201 (96.7)		
Group 2, *n* (%)	9900 (36)	521 (5.3)	9379 (94.7)		
Group 3, *n* (%)	1730 (6)	135 (7.8)	1595 (92.2)		

^1^ Formal ICD-10 diagnosis determined by a physician in hospital care or during an outpatient visit. ^2^ Mosaic groups = socioeconomic groups at the area level, where group 1 is the most and group 3 is the least advantaged group. ^3^ HFRS = Hospital Frailty Risk Score: group 1 ≤ 5 points (low risk of frailty), group 2 = 5–15 points (intermediate risk), and group 3 ≥ 15 points (high risk).

**Table 2:** Depression in relation to cancer type.

Rank	Cancer Type, *n*	Depression (%)
1	Hematologic malignancies, *n* = 3655	5.1
2	Brain tumor, *n* = 1094	4.9
3	Lung cancer, *n* = 4110	4.4
4	Gynecologic cancer, *n* = 1432	4.3
5	Head and neck cancer, *n* = 1099	4.3
6	Prostate cancer, *n* = 2372	4.3
7	Colo-rectal cancer, *n* = 2400	3.9
8	Pancreatic cancer, *n* = 2384	3.9
9	Breast cancer, *n* = 1437	3.6
10	Others, *n* = 7360	4.1
	**Total, *n* = 27,343**	**4.3**

**Table 3:** Health care needed and depression in the last year of life.

Variable	Depression (*n* =1168)	No Depression (*n* = 26,175)	Chi-Square (DF)	*p*-Value
Emergency room visits ^1^, *n* (%)	1059 (91)	22,549 (86)	19.4 (1)	<0.001
Visits in psychiatric services ^1^, *n* (%)	366 (28)	923 (3)	1925.0 (1)	<0.001
Intravenous cancer treatment ^1^, *n* (%)	422 (36)	12,220 (47)	50.1 (1)	<0.001
Specialized palliative care ^1^, *n* (%)	895 (77)	20,230 (77)	0.3 (1)	0.60
Emergency hospitals as place of death	205 (18)	4750 (18)	0.3 (1)	0.60

^1^ One or more visits/admissions/treatments during the last year of life.

**Table 4:** Univariable and multivariable regression analyses.

Variable	Number (%) *n* = 27,343	Univariable		Multivariable *	
		OR ^1^ (95% CI) ^2^	*p*-Value	aOR ^3^ (95% CI)	*p*-Value
Age groups					
18–79 years	19,362 (71)	Ref.		Ref.	
≥80 years	7951 (29)	1.14 (1.01–1.30)	0.04	1.02 (0.90–1.16)	0.76
Sex					
Men	14,015 (51)	Ref.		Ref.	
Women	13,328 (49)	1.26 (1.12–1.41)	<0.0001	1.30 (1.16–1.46)	<0.001
HFRS ^4^: frailty risk					
Low	15,713 (58)	Ref.		Ref.	
Intermediate	9900 (36)	1.65 (1.46–1.87)	<0.0001	1.66 (1.46–1.88)	<0.001
High	1730 (6)	2.51 (2.01–3.06)	<0.0001	2.57 (2.10–3.14)	<0.0001

* c-statistic was 0.59, and the Hosmer and Lemeshow goodness of fit test was 0.76. ^1^ Odds ratio. ^2^ 95% confidence interval. ^3^ Adjusted odds ratio. ^4^ HFRS = Hospital Frailty Risk Score: group 1 ≤ 5 points (low risk of frailty), group 2 = 5–15 points (intermediate risk), and group 3 ≥ 15 points (high risk).

## Data Availability

The datasets generated and analyzed in this study are available on reasonable request.

## Abbreviations

The following abbreviations are used in this manuscript:

DSM	Diagnostic and Statistical Manual of Mental Disorders
ER	Emergency room
HFRS	Hospital Frailty Risk Score
ICD-10	International Statistical Classification of Diseases and Related Health Problems, Tenth Revision
SPC	Specialist palliative care
